# Community-associated Methicillin-resistant *Staphylococcus aureus*, Colombia

**DOI:** 10.3201/eid1212.060814

**Published:** 2006-12

**Authors:** Carlos A. Alvarez, Oscar J. Barrientes, Aura L. Leal, German A. Contreras, Liliana Barrero, Sandra Rincón, Lorena Diaz, Natasha Vanegas, Cesar A. Arias

**Affiliations:** * Hospital Simón Bolívar, Bogotá D.C., Colombia;; † Universidad Nacional, Bogotá D.C., Colombia;; ‡ Universidad El Bosque, Bogotá D.C., Colombia;; § University of Texas Medical School at Houston, Houston, Texas, USA

**Keywords:** MRSA, Colombia, Methicillin-resistant *Staphylococcus aureus,*

To the Editor: Methicillin-resistant Staphylococcus aureus (MRSA) is an established nosocomial pathogen worldwide but more recently has emerged as a highly virulent organism in the community, particularly in the United States (*1**–**3*). In Latin America, community-associated MRSA (CA-MRSA) has only been described in the southern area of the continent (Uruguay and Brazil) (*4**,**5*). No reports from the Andean region are available. We describe 2 cases of CA-MRSA causing soft-tissue infections (1 severe) in Colombia.

The first case was in a 19-year-old man with a history of trauma to the left side of his body 1 week before admission after a fall. On admission, he complained of 2 days of fever, malaise, erythema and induration in the left hemithorax extending to the left thigh, and purulent secretion from an excoriation on the anterior aspect of the left thigh. He had no previous medical history. No previous hospitalizations or antimicrobial drug prescriptions were documented, nor did he report relatives with history of recent hospitalizations. Vital signs at admission were normal except for fever (39°C), and physical examination showed induration and erythema in the region of left hemithorax extending to the thigh, with an area of excoriation in the same thigh with purulent discharge. Laboratory evaluation showed a leukocyte count of 23.1×10^9^/L (86% neutrophils with 2% band forms) and elevated C-reactive protein levels.

The patient was hospitalized. Because necrotizing fascitis was suspected, intravenous ampicillin-sulbactam (12 g per day) was started, and surgical evaluation was requested. The patient underwent surgical debridement of the left thigh, left hemiabdomen, and hemithorax, which confirmed the diagnosis of necrotizing fascitis. Intravenous vancomycin (1 g every 12 h) was added to the regimen, and the patient was transferred to the intensive care unit. After several surgical debridements, the patient underwent placement of cutaneous-muscle grafts. He was discharged from the hospital without complications after completing 14 days of antimicrobial agents.

The second case involved a 53-year-old man with no history of previous hospitalizations who reported to the emergency department with a 3-day history of fever, pain, swelling, and warm sensation on the posterior aspect of the left thigh. A diagnosis of cellulitis was made, and cephalexin (500 mg every 6 h) and gentamicin (160 mg intramuscularly every 24 h) were administered for 7 days without improvement. He returned to the hospital with worsening symptoms, an area of induration of 4×4 cm in the thigh, and purulent discharge. Drainage of the lesion was performed, and oral trimethoprim and sulfamethoxazole (160 and 800 mg, respectively, every 12 h) was started. His clinical signs and symptoms completely resolved after 7 days of therapy.

Tissue culture from secretions from both patients showed gram-positive cocci in clusters on the Gram stain, and subsequent cultures yielded MRSA. Species identification and presence of the mecA gene were confirmed by PCR, as described previously (*6*). MICs were determined by using the agar diffusion test, according to Clinical and Laboratory Standards Institute recommendations (*7*). Both organisms were susceptible to vancomycin, teicoplanin, chloramphenicol, linezolid, ciprofloxacin, gentamicin, and rifampin. The isolate from the second patient was resistant to erythromycin and susceptible to clindamycin, exhibited the M phenotype on the double-disk diffusion assay (D test), and harbored the msrA gene, encoding an efflux pump (*8*). In contrast, the first isolate was susceptible to both erythromycin and clindamycin and resistant to tetracycline (MIC >64 μg/mL). Because infections caused by CA-MRSA isolated elsewhere are associated with the presence of the lukF gene encoding the Panton-Valentine leukocidin toxin and the staphylococcal chromosome cassette mec (SCCmec) type IV, the presence of both was evaluated by PCR, as described previously (*9*). Both isolates were positive for lukF and harbored the SCCmec type IV.

The molecular epidemiology of healthcare-related MRSA in Colombia has changed during the past 3 years (*10*), but no reports of CA-MRSA had emerged. We believe these to be the first reports of CA-MRSA in Colombia with similar characteristics to those reported elsewhere. No risk factors associated with healthcare-associated MRSA were found in either of these patients, and the patients were not epidemiologically related. The first case involved a severe soft-tissue infection associated with CA-MRSA. Clinicians should be aware of the circulation of CA-MRSA in Colombia.
